# Femtosecond Laser Machining of an X-ray Mask in a 500 Micron-Thick Tungsten Sheet

**DOI:** 10.3390/mi14112071

**Published:** 2023-11-07

**Authors:** Ebenezer Owusu-Ansah, Colin Dalton

**Affiliations:** Department of Electrical & Software Engineering, Schulich School of Engineering, University of Calgary, 2500 University Drive NW, Calgary, AB T2N 1N4, Canada; cdalton@ucalgary.ca

**Keywords:** ultrafast lasers, laser ablation, ultrafast laser processing, femtosecond laser pulse, femtosecond laser material processing (FLMP), computer numerically controlled (CNC) motion, CAD/CAM, tungsten X-ray mask

## Abstract

Femtosecond laser material processing (FLMP) was used to make an X-ray mask in a 500 µm thick tungsten sheet without the use of any chemical etch methods. The laser produced an 800 nm wavelength at a 1 kHz repetition rate and a pulse width of 100 fs. The laser beam arrival at the tungsten sheet was synchronized to a computer numerically controlled (CNC) stage that allowed for motion in the XYZθ directions. The X-ray mask design was made using CAD/CAM software (Alphacam 2019 R1) and it consisted of linear, circular, and 45° angle features that covered an area of 10 mm × 10 mm. A total of 70 laser beam passes at a moderate laser energy of 605.94 J/cm^2^ were used to make through-cut features into the tungsten sheet. The morphology of the top view (laser incident, LS) images showed cleaner and smoother cut edges relative to the bottom view (laser exit, LE) images. It was found that the size dimensions of the through-cut features on the LE surfaces were better aligned with the CAD dimensions than those of the LS surfaces. The focused laser beam produced inclined cut surfaces as the beam made the through cut from the LS to the LE of the tungsten sheet. The circular features at the LS surface deviated toward being oval-like on the LE surface, which could be compensated for in future CAD designs. The dependence of the CNC processing speed on the thickness of the etch depth was determined to have a third-order exponential decay relationship, thereby producing a theoretical model that will be useful for future investigators to predict the required experimental parameters needed to achieve a known etch depth in tungsten. This is the first study that has demonstrated the capability of using a femtosecond laser to machine through-cut an X-ray mask in a 500 µm thick tungsten sheet with no involvement of a wet etch or any other such supporting process.

## 1. Introduction

Tungsten is a heavy absorbing material due to its high atomic number (74) and high density (19.3 g/cm^3^), which is comparable to gold (79, 19.3 g/cm^3^) [1,2,3]. Though a ~205 µm thick tungsten is needed to absorb ~90% of an incoming 50 keV X-ray beam relative to a piece of 167 µm thick gold, the cheaper material cost of tungsten has made it an attractive substitute for high-energy radiation-absorbing applications, including X-ray masks [4,5,6,7]. Other high-energy-absorbing metals, such as iridium and platinum, are expensive in comparison to tungsten. Several processing techniques have been explored for creating features in tungsten. Chemical etch methods are known to suffer from isotropic etch profiles, i.e., uniform material removal in all directions, which is known to cause undercutting, thus making it difficult to control lateral feature size resolution [8] and achieving channels with high aspect ratios [9]. Atomic layer etching techniques, where atomic layers are sequentially removed, are one type of possible solution that have been reported [8,10,11]. However, these methods all depend on the use of fluorine-containing chemicals, a hazardous chemical that requires careful handling and disposal. Similarly, reactive ion etching (RIE) or ion-assisted plasma etching, a technique that combines the radical chemical-based process and ion bombardment, can be controlled to produce either unidirectional (anisotropy)—which is responsible for producing vertical features—or multi-directional (isotropy) [12,13]. Unlike femtosecond laser material processing (FLMP), RIE is a sophisticated process that requires the use of reactive, dangerous, and hazardous chemicals. Focused ion beam (FIB) is another method that has been used for high-precision material processing [14,15], including metals such as tungsten [16]. In this method, ions generated from argon or gallium atoms are extracted and focused by an electric field onto the target substrate. Material surface modification occurs by the interaction of the high-energy ions with the substrate through implantation and the sputtering of atoms [14]. In comparison to FIB, FLMP has a relatively higher removal rate, approximately 4–6 orders of magnitude better than FIB, with minimal substrate damage [16,17]. Here, we avoid the challenges of tungsten fabrications, which are associated with all the above methods, by using FLMP to produce a tungsten X-ray mask with linear and circular geometries that would otherwise have been very challenging to achieve through conventional methods.

The uniqueness of the FLMP method is the ability to achieve the laser ablation of material substrates with no or minimal thermal damage. When a femtosecond (fs) laser pulse (10^−12^ s) incidents on a substrate material, the timespan for photon absorption by the substrate occurs at a timescale of ~10^−14^–10^−13^ s, which is shorter than the time required for electron–phonon coupling relaxation processes (10^−12^–10^−11^ s), thus ensuring energy is delivered to the electrons while the ions and the lattice are left “cold”. Hence, within the duration of the fs pulse, there was minimal to no thermal energy transfer to the lattice, thus decoupling the optical absorption processes from the lattice heat transfer processes [18]. Fs pulses produce high-peak electric fields (~10^12^ V/m) that are ~3 orders of magnitude greater than the electric field (10^9^ V/m) that binds electrons to atoms. When the fs laser beam is tightly focused, the high-peak electric field allows for non-linear optical absorption processes to occur, e.g., multiphoton absorption and tunneling ionization [19,20]. Multiphoton absorption allows excited electrons to acquire enough photon energy to cause ionization and bond breakage, which is followed by material removals from the substrates that have minimal heat-affected zones (HAZs). Several experimental and theoretical studies have been conducted to understand the mechanism involved in ultrafast laser ablation [21,22,23,24]. Hernandez-Rueda et al. used both theoretical and experimental approaches to show the gradual loss of mass from gold nanoparticles, which occurred following exposure to fs laser pulses [22]. Jia and Zhao recently reported a theoretical numerical study to explore the optimal conditions through which to achieve high material removal, as well as to increase laser ablation efficiency when an ultrafast laser pulse incidents on a copper substrate [25]. These investigations underscore the importance of the fs laser ablation method, and a better understanding of this technique may reveal latent future applications.

Reports on FLMP to machine tungsten substrates includes the work of Pfeifenberger et al., who machined an array of cantilevers into tungsten foils of ≤100 µm thick [16]. As previously mentioned, such foil thickness is below the required tungsten thickness needed for use in high-energy absorption applications, such as X-ray masks. Silvestre et al. used a picosecond laser to make through-hole arrays in a 200 µm thick tungsten sheet for use as an X-ray optical grating [4]. To achieve the desired hole dimension, the group used an additional wet chemical etch process composed of aqueous ammonia and hydrogen peroxide to widen the holes; this also assisted with the removal of re-deposited tungsten debris. In this work, we used FLMP to successfully make an X-ray mask by creating through-cut features of different geometries into a 500 µm thick tungsten sheet while avoiding the use of any wet chemical etch processes and their associated health hazards. To the best of our knowledge, our work here is the first to report on using FLMP to make X-ray masks in a 500 µm thick tungsten sheet with no wet chemical etch or other such support processes.

## 2. Materials and Methods

**Figure 1:**
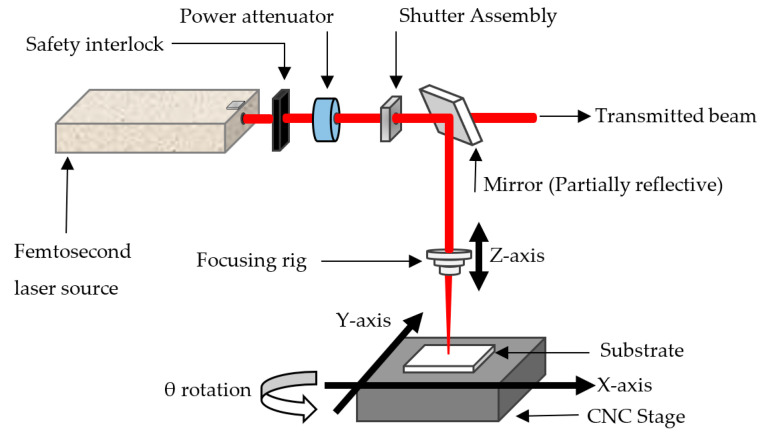
A simplified schematic showing the experimental setup used in the FLMP of a 0.5 mm thick tungsten sheet. The angle of incidence of the focused laser beam was perpendicular to the tungsten sheet surface.

A description of the experimental setup has been published elsewhere [26], and a brief description is given here. Figure 1 shows a simplified schematic description of the FLMP experimental setup. The femtosecond laser source is composed of a mode-locked seed laser—the Mai Tai^®^ SP Ti:Saphire, a pump laser—an intercavity-doubled diode-pumped Empower Nd:YLF laser, and a regenerative amplifier (the Spitfire PRO Ti:Saphire laser system (Spectra-Physics, Milpitas, CA, USA). The laser produces a tunable infra-red (IR) radiation in the wavelength region of 780–820 nm and a variable repetition rate from 0.1–1 kHz. A central wavelength of 800 nm and a pulse width of 100 fs was used in this study unless stated otherwise. The laser was synchronized to a computer numerically controlled (CNC) stage (Aerotech, Inc., Pittsburgh, PA, USA) that allows for motions in the XYZθ directions. The laser beam was directed through a set of optical components, such as a shutter assembly (Uniblitz, Rochester, NY, USA), energy attenuator, and a safety interlock. During FLMP, the laser was always on; hence, the shutter was used to block off the laser beam when no machining was required, especially when the CNC stage was moving to machine a feature at a different location, thus preventing laser scoring on the substrate surface. The energy attenuator was used to control the amount of laser energy arriving at the material substrate. One of the mirrors was partially reflective, thus enabling a small amount of the laser beam to be transmitted and fed into a power meter for the continuous monitoring of laser energy.

**Figure 2:**
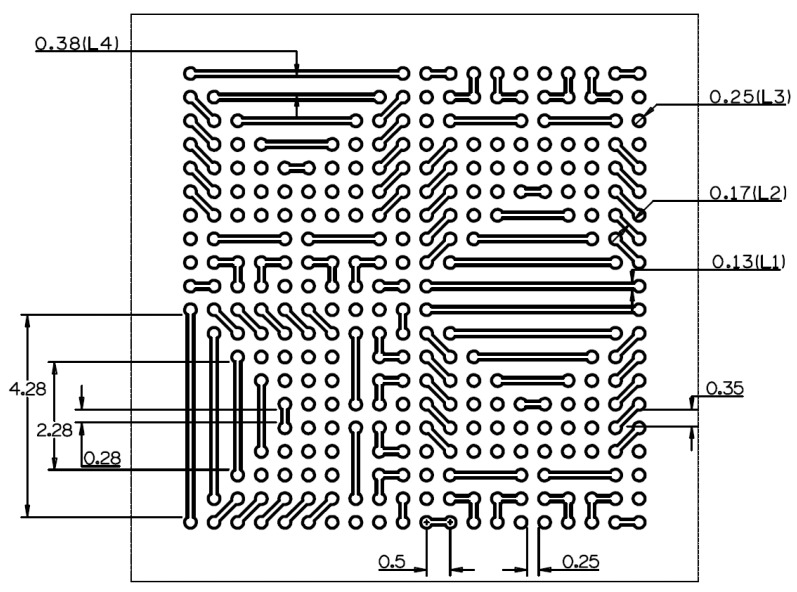
A 2D top view of a CAD schematic representation of an X-ray mask to be machined in a 0.5 mm thick tungsten substrate. Dimensions are in mm units. The width of the channels, called channel throat, was 0.13 mm, while the distance between the two channel features, called pore body, was separated at lengths ranging from 0.17–0.38 mm. The diameter of all of the circular features was 0.25 mm. Notations such as (L1) and (L2) simply denote the dimensions where there were comparison data on the CAD, laser incident surface side (LS), and laser exit (LE). See a further discussion in Table 1.

A tungsten sheet, 0.5 mm thick (on a 99.95% metals basis), was purchased from Alfa Aesar^®^ (Haverhill, MA, USA) and 25 mm × 25 mm pieces were used. A lens with a focal length of f=200 cm was housed in a focusing rig and attached to the Z axis of the CNC motion stage, thus allowing for the laser beam to be re-focused at different depths of the tungsten substrate. This was used when multiple laser beam passes were required to machine the desired through-cut features in the 0.5 mm thick tungsten sheet. The desired pattern shown in Figure 2 was made in CAD/CAM software (Alphacam, 2019 R1, Hexagon, Stockholm, Sweden). The design is composed of mostly circular and linear features that cover approximately a 10 mm × 10 mm surface area. The design consists of 0.25 mm circles that are surrounded by predominantly linear features with a dumbbell-like design of varying length. The linear features have several orientations, including vertical, horizontal, and a 45° angle. For simplicity, these linear geometries will be referred to as channels. A few L-shape designs were also present. It is worth noting that the majority of these features occurred multiple times across the design. This was to allow for studies on the repeatability of the FLMP to be conducted. Femtosecond laser pulses have been shown to have less HAZ in comparison to pico- and nanosecond laser pulses [18,27]. To further minimize HAZ, as well as to reduce the potential for cracks and rough feature edges, a moderate laser power of 0.794 W was used, which is equivalent to an energy density (fluence) of 605.94 J/cm^2^ when based on a laser-focused beam diameter of 12.3 µm [26]. At a processing speed of 1 mm/s, a total of 70 laser beam passes were required to achieve complete through-cut features. Following each laser beam pass, the next laser beam was re-focused to the cut surface by moving the Z axis downward by ~7.1 µm.

Profiles of the FLMP machined features were measured with a contact profilometer (Dektak XT, Bruker, MA, USA), while images were taken with a scanning electron microscopy (SEM) (Phenon XL, ThermoFisher, Waltham, MA, USA). Image J software (NIH, Ver. 1.52a) was used for the dimension analysis of the SEM images.

## 3. Results and Discussion

### 3.1. Surface Morphology of an FLMP-Machined Tungsten Sheet

**Figure 3:**
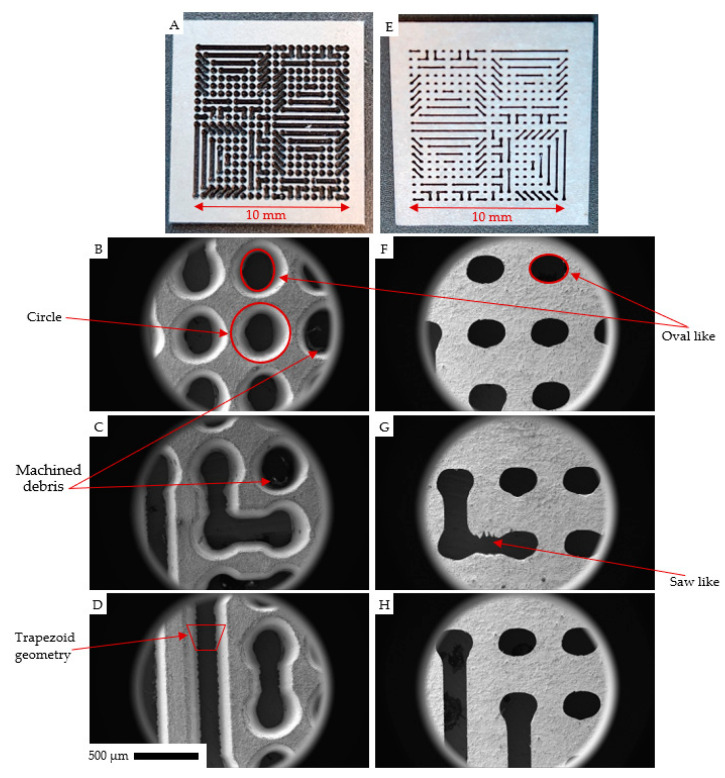
(**A**–**D**) The top view images (laser incident (LS) surface). (**E**–**H**) The bottom view images (laser exit (LE) surface) of the FLMP X-ray mask. A and E are the optical camera images that show the total area view, while the rest of the images (**B**–**D**,**F**–**H**) were taken with an SEM that showed a smaller area view. Images (**A–D**) are representative of top view and (**E**–**H**) are representative of bottom view images. The arrows are pointing to morphological regions of interest, including circles, ovals, saw-like shapes, and machined debris. The trapezoid geometry in (**D**) illustrates the cross-section of the channel, where the long and short base lines represent the LS and LE length, respectively. The scale bar on (**D**) is for all images except (**A**,**E**).

Figure 3 shows the SEM and optical images of different sections of the FLMP-machined tungsten sheet. Images (A–D) were taken from the laser incident (LS) surface (‘top’ of the tungsten), while (E–H) were taken from the laser exit (LE) surface (the ‘bottom’ side). Images A and E were optical camera images, while the rest of the images (B–D,F–H) were taken with an SEM. Generally, all of the SEM images showed fine through-cut features, but the LS surface had finer cut edges relative to the LE surface. For instance, the cut-edge portions of Figure 3G show a saw-like cut edge, while none was observed for the LS surface. We attribute this to the inherent brittle nature of tungsten, which makes the cut-edge portions, such as the channel throat, become more susceptible to substrate breakage due to localized sample agitations from the multiple laser beam passes. To minimize this saw-like edge-cut morphology at the LE surface, a relatively lower laser power and a slower processing speed would be required relative to what was used in this work.

However, that would also prolong the processing time and hence a trade-off decision would need to be made. Another point of interest is the apparent deviation of the circular features into oval-like shapes. There is very minimal to nonexistent evidence of this on the LS surfaces in Figure 3B–D, but they are significantly enhanced in the LE surface images in Figure 3F–H. This is highlighted with the red circle and red oval-like shapes drawn on top of Figure 3B,F, respectively. It was noted that the oval-like features exhibited in the LE surface images can also be seen at the bottom of the LS surface images, as denoted by another oval-like shape at the bottom of Figure 3B. Here, we attribute the oval-like shape morphology to two factors, i.e., the shape of the laser beam due to the optical path travel, and the thickness of the substrate. The shape of the focused laser beam arriving at the substate surface as it progresses deeper into the material with each successive material removal is likely skewed toward producing oval-like shapes due to the longer travel path, which consists of both horizontal and vertical optical paths. The LS morphology favors circular features. However, as the beam makes its way through the 0.5 mm thick tungsten substrate, any slight skewness in the laser beam shape (i.e., the un-equal power distribution) is expected to reflect sharply in the shape of the cut feature due to the pockets of un-etched substrate that prevent successive laser–substrate interactions. This can be accounted for in future CAD designs to compensate for the observed skewness and oval-like morphologies at the LE surface. Figure 3B,C have arrows pointing to the presence of the minimal residual debris. Here, in this study, a vacuum exhaust system was introduced to remove the machined debris, and this worked well for the through-cut X-ray mask, thereby requiring no further wet etch treatment.

### 3.2. Cut-Edge Morphology of FLMP-Machined Tungsten Sheet

**Figure 4:**
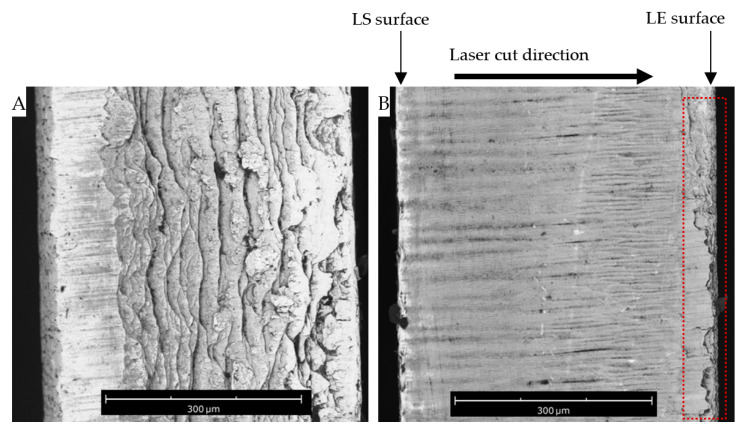
SEM images taken at 90° to the cut surface of the (**A**) as-purchased tungsten sheet in comparison to that of the (**B**) FLMP cut surface. The LS surface and LE surface arrows point to the laser incident and laser exit regions across the 0.5 mm thick tungsten sheet. The laser cut direction is from the LS to the LE surfaces, as indicated by the cut direction arrow. The LS region had a smooth cut edge relative to the LE region (dashed rectangle).

The quality of the cut edge was examined by SEM images at a 90° angle to the laser cut surface by using a vertical mounting stud. Figure 4 shows the cut edge of the as-purchased tungsten sheet in comparison to the FLMP cut edge. The as-purchased cut edge shown in Figure 4A has a very rough cut-edge morphology relative to the FLMP cut edge. Though there is not enough information on the method used to cut the as-purchased tungsten sheet, the overall cut-edge morphology observed for the FLMP in Figure 4B shows a smooth cut-edge quality relative to that of the as-purchased tungsten sheet.

It is worth mentioning that the cut edge was smoother at the LS surface, but the quality reduced as it moved through the 0.5 mm thick tungsten substrates to the LE surface, thus leaving the LE surface region, as shown in the dashed rectangle of Figure 4, with an increased surface roughness. This observation corroborates the previous section of this study, where the LE surfaces were found to be relatively less smooth and had, in some cases, saw-like features in comparison to the LS surfaces, which had clean and smooth cut edges. These observations agree with a previous report that studied the cut-edge quality of material substrates following fs laser machining. Edgaras et al. used a 1030 nm fs laser to make through-cut features into a borosilicate glass in air and water environments. They obtained optical micrographs on the cut edges of the borosilicate glass, and they found that the top view cut edge (LS surface) was smoother and cleaner in comparison to the bottom view cut edge (LE surface) for both environments [28].

The use of fs laser pulses to ablate material substrates has been shown to cause oxidation, which can be controlled for other useful applications [19,29,30]. Most of these investigations, including the experimental setup used for this study, were conducted in an air environment; hence, there is the potential for oxidation process exits when using fs lasers to ablate material substrates. Zhou et al. used a 1030 nm fs laser to ablate SiC composites, and they characterized the ablated surfaces using several techniques, such as energy dispersive spectroscopy (EDS), X-ray diffractometry (XRD), X-ray photoelectron spectroscopy (XPS), and Raman spectroscopy [19]. The group observed SiO_2_ and CO as the main oxidation products that were produced by the laser ablation of the SiC composites, and they determined the experimental conditions that could be used to control the degree of oxidation. Florian et al. used a 790 nm fs laser pulse at a 1 kHz repetition to ablate a thin layer of chromium nitride (CrN) on a steel substrate, and this was followed by a comprehensive material characterization using EDS, XRD, SEM, atomic force microscopy (AFM), and variable angle spectroscopic ellipsometry (VASE) [30]. The group also performed theoretical simulations using the finite-difference time-domain (FDTD) method. The group observed the production of a laser-induced oxide that agreed with their theoretical simulations. A recent report by Haase et al. showed that tungsten oxide (WO_3_) nanoparticles and aggregate structures were formed through direct fs laser pulse ablation on WO_3_ substrates in an air environment [31]. The group used a 1030 nm fs laser at a 300 kHz repetition for their experiment. However, the presence of oxide in the substrate prior to laser ablation made it difficult to conclude that the fs laser ablation process caused the oxide formation in the produced nanoparticles and aggregate structures. Here, in this study, the fs laser was used to produce a tungsten X-ray mask; hence, the presence of oxide formation should not have an impact on the desired application. Nonetheless, a future work on the characterization of the ablated tungsten surface to determine if the ablation process produces tungsten oxide could provide more insights for future researchers regarding the fs laser ablation of tungsten, as well as possibly for other surface chemistry applications.

### 3.3. Characterization of the FLMP-Machined Feature Sizes

An analysis of the feature size dimensions was made using Image J software, and the results are shown in Table 1. Following the FLMP of the tungsten sheet, it was observed that the through-cut features measured from the LS surface, represented by the channel throat (L1) and the circle (L3), generally became wider than the size defined by the CAD dimension. For example, the channel throat (L1) dimension at the LS surface was 316.2 ± 3.5 µm, thus producing a ratio of 2.4 relative to the CAD dimension. Similarly, the other through-cut feature, the circle (L3), showed that the feature size of the LS increased relative to the CAD size dimension with a corresponding LS/CAD ratio of 1.8. This was largely attributed to the kerf–burnt portion of a material when laser cuts through, via the multiple laser beam passes (70 total passes), to achieve a successful through cut for the 0.5 mm thick tungsten sheet.

The observed oval-like morphology of the LE surface (L3) was supported by an average diameter size of 262.7 µm with a wide standard deviation of ±38.3 µm. During the diameter size determination, the major and minor axis diameters were both extracted and averaged together. In comparison to the LS surface, the average diameter was 461.3 µm, with a modest standard deviation of ±6.2 µm. The LS/CAD ratios of the through-cut features of L1 and L3 were 2.4 and 1.8, while the LE/CAD ratios were 1.4 and 1.1, respectively. This implied that the features sizes of the LE surface (bottom view dimensions) aligned more closely with the CAD dimensions than that with the LS surfaces (top view dimensions). This was expected since the kerf of the focused laser beam impacts the LS surface more than the LE surface dimensions. The measured size dimensions in Table 1, in particular the channel throat (L1) and the circle (L3) feature sizes, illustrate a similar symmetrical trapezoid geometry to that depicted in Figure 3D. Here, the calculation of the size dimensions show that the inclined cut surfaces make contact angles (θ) of ~8 and ~11° with a vertical plane for the channel throat (L1) and circle (L3) geometries, respectively (when considering the LS surface images). The contact angle of ~11° for the circular through-cut features was found to be slightly larger than ~8°, and this was primarily due to the error introduced by the wide deviation in the major and minor diameter size determinations. Along the path of this inclined angle, ~90% of an incoming 50 keV X-ray beam can be absorbed only when the tungsten thickness is ~205 µm. Knowing this angle of inclination allowed for the determination of the effective width (W_E_), at which the tungsten thickness was ~205 µm. At a 205 µm thickness, which was measured from the LE surface, the W_E_ of the through-cut channel throat (L1) was 233.3 µm. For the channel throat L1 in Table 1, the ratio of this effective width relative to the CAD, LS, and LE dimensions were 1.8, 0.7, and 1.3, respectively. Hence, consideration should be given to the effective width during the designing stage to ensure the desired width is achieved following the FLMP of a tungsten sheet for X-ray absorption applications.

### 3.4. Dependence of the Etch Depth on CNC Processing Speed

**Figure 5:**
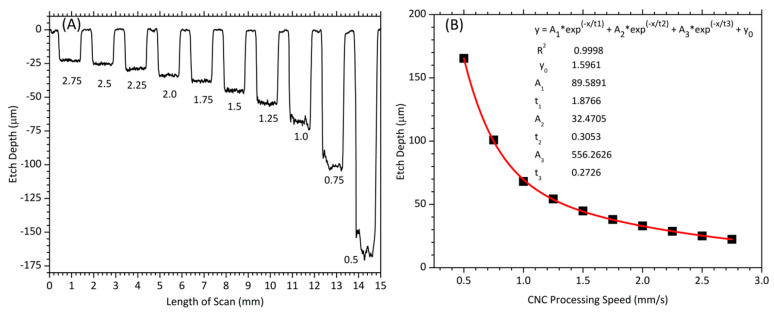
(**A**) A line profile scan across multiple FLMP-etched depths into tungsten sheet at a constant fluence of 353.47 J/cm^2^ and at different CNC processing speeds. A pitch of 5 µm was used, and the different speeds in mm/s are shown below each depth. (**B**) An exponential decay fitting based on measured experimental data points (black squares) as a function of the CNC processing speed. The theoretical fitting results are given on the graph.

The dependence of the etch depth on the CNC processing speed was explored by etching 1 mm × 1 mm square features on a tungsten sheet at a 353.47 J/cm^2^ fluence and 5 µm pitch (center-to-center of the nearness laser beam pass) while varying the speed. Figure 5 shows a line profile scan and a theoretical curve fitting plot.

The line profile scan in Figure 5A shows that the CNC processing speeds ranging from 2.75–1.25 mm/s produced smooth etch surfaces relative to the profiles at speeds of 1.0–0.5 mm/s. This was primarily attributed to the competition between the buildup of debris and the incident laser beam, which obstructed the laser beam from reaching a pristine surface. This has been observed by other investigators [32,33,34], especially for deeper channels as they suffer the most from reduced debris removal and subsequent interference with the incoming incident laser beam. Here, the vacuum exhaust used worked well for the X-ray mask for the majority of the multiple etched depths shown in Figure 5, but debris still caused significant surface roughness to the deeper channels with depths of >100 µm. The aim of the curve fitting was to explore a theoretical fitting model that best fit the experimental data so that the fitting can serve as guiding tool to aid future researchers to determine the experimental parameters needed to produce a known etch depth. From this exercise, a third-order exponential decay function was determined to be the best fitting model with an R^2^ value of 0.9998.

## 4. Conclusions

Tungsten is an attractive material for use in high-energy absorption applications, such as X-ray masks; however, it is brittle and challenging to process through conventional methods. In this study, femtosecond laser material processing (FLMP) was used to machine through-cut features into a 0.5 mm thick tungsten sheet to create an X-ray mask. The machined X-ray mask covered a 10 mm × 10 mm area, and it consisted of linear and circular features. The SEM characterization of the machined features showed that the feature size dimensions of the laser exit surface (LE) were better aligned with the CAD dimensions relative to the laser incident surface (LS). The cut edge of the tungsten sheet that was cut by the FLMP was smoother and had a better quality finish than that of the as-purchased tungsten sheet. The through-cut surface from the top to the bottom of the tungsten sheet made a contact angle of ~8 and ~11° with the vertical plane for the channel throat (L1) and circle (L3) geometries, respectively. To effectively stop ~90% of incoming 50 keV X-ray beams, the effective width (W_E_), at which the tungsten thickness of the channel throat (L1) was ~205 µm, was determined to be 233.3 µm, thus producing a W_E_/CAD ratio of 1.8. The dependence of etch depth was determined to have a third-order exponential decay dependence on the CNC processing speed. This study has shown the capability of using FLMP to produce linear and circular geometries into tungsten sheets, which could be useful in aiding future developments of complex designs in tungsten substrates for high-energy radiation absorption applications.

## Figures and Tables

**Table 1:** Comparison of the feature sizes among the CAD dimensions, and among those of the laser incident (LS) and laser exit (LE) surface dimensions. The L1–L4 notation refers to the feature type where data were collected, as shown in the CAD in Figure 2.

Feature Description	CAD (µm)	LS (µm)	LE (µm)	Ratio (LS/CAD)	Ratio (LE/CAD)	Ratio (LS/LE)
L1, channel throat	130	316.2 ± 3.5	182.3 ± 2.2	2.4	1.4	1.7
L2, pore body	170	98.7 ± 2.9	255.1 ± 1.3	0.6	1.5	0.4
L3, circle	250	461.3 ± 6.2	262.7± 38.3	1.8	1.1	1.8
L4, pore body	380	176.7 ± 2.5	312.0 ± 2.6	0.5	0.8	0.6

## Data Availability

Data is contained within the article.
